# Effects of epidural anesthesia and analgesia on the incidence of chronic pain after thoracoscopic lung surgery: A retrospective cohort study

**DOI:** 10.1016/j.heliyon.2024.e35436

**Published:** 2024-07-31

**Authors:** Yiming Liu, Chenyu Wang, Zhixiang Ye, Ling Jiang, Changhong Miao, Chao Liang

**Affiliations:** a Department of Anesthesiology, Zhongshan Hospital, Fudan University, Shanghai, China; b Department of Anesthesiology, Zhongshan Hospital (Xiamen), Fudan University, Xiamen, China

**Keywords:** Thoracic surgery, Chronic postoperative pain, Thoracic epidural anesthesia, Video-assisted thoracoscopic surgery, Patient-controlled intravenous analgesia

## Abstract

**Objective:**

Chronic postoperative pain (CPSP) is common after thoracic surgery, even after the less invasive video-assisted thoracoscopic surgery (VATS). This study investigated the effect of thoracic epidural anesthesia (TEA) on the development of CPSP.

**Materials:**

We retrospectively analyzed the data of patients who underwent VATS at our center between 2020 and 2022. The enrolled patients were divided into the epidural block (EPI) and patient-controlled intravenous analgesia (PCIA) groups. A telephone questionnaire was used to collect information regarding CPSP, which was defined as a numerical rating scale (VAS) score ≥1 at 3 or 6 months postoperatively. Additionally, statistical analyses were performed to identify the risk factors for CPSP in the two groups.

**Results:**

Overall, 894 patients completed the follow-up interviews at 3 and 6 months, with 325 and 569 patients in the PCIA and EPI groups, respectively. The incidence rates of CPSP in the PCIA group at 3 and 6 months were 16.9 % (95 % confidence interval [CI]: 9.3–32.7 %) and 13.5 % (95 % CI: 8.7–33.4 %), and 10.3 % (95 % CI: 8.1–30.5 %) and 3.6 % (95 % CI: 3.5–21.5 %) in EPI group, respectively. The incidence of CPSP at 3 months (P = 0.0048) and 6 months (P < 0.005) was statistically significant in both groups. Age and lymph node dissection were significantly associated with CPSP.

**Conclusions:**

Compared to PCIA, TEA was associated with a lower incidence of CPSP after VATS, and should be considered an important part of the analgesia regimen for patients with VATS.

## 1 Introduction

Chronic postsurgical pain (CPSP) has been defined by the International Association for the Study of Pain as pain that persists for > 3 months [1]. It adversely affects the quality of life of patients and delays rehabilitation and return to usual activities. Despite the advances in medical care, no significant reduction in the incidence and severity of CPSP has been achieved [2].

Thoracic surgery is a major surgery that is commonly performed to manage various diseases or injuries, such as lung cancer, lung transplantation, pleural cancer, lung emphysema, post-trauma abscesses, or pleurodesis for recurrent pneumothorax [3]. Thoracic surgery is one of the most painful procedures [4]. Despite the improvements in surgical techniques and pain management interventions, CPSP has an incidence ranging 14–83 % [5], and is one of the most common complications of thoracic surgery [6,7]. Video-Assisted thoracoscopic surgery (VATS) is considered less invasive than open thoracotomy, with less likelihood of causing nerve injury. However, accumulating data indicate that VATS and thoracotomy have similar rates of chronic pain, which may be related to intraoperative damage to the nerves and myofascial structures, as well as the prolonged duration of postoperative chest tube drainage [8].

Inadequate postoperative pain management is a major risk factor of CPSP [9]. Recent recommendations from the Society of Cardiovascular Anesthesiologists (SCA) suggest that perioperative thoracic epidural analgesia (TEA) should be used for acute pain management after open thoracotomy and that TEA can be also used for acute pain management after VATS [9]. However, the recent PROSPECT group recommendations [10] do not recommend TEA, even as a second-line option for VATS; their main concerns are epidural-related hypotension, urinary retention, difficulties in catheter insertion, or drug injection errors with serious consequences. Additionally, TEA is more invasive compared to other regional procedures [10]. However, even the continuous analgesia technique of paravertebral space catheterization cannot replace continuous epidural analgesia after VATS, and the epidural technique is still considered the gold standard [11]. However, few studies have explored the association between TEA and CPSP after VATS.

Considering the effective pain management provided by TEA, we speculated that it might also decrease the incidence of CPSP after VATS. Therefore, the objective of this retrospective study was to investigate whether the application of TEA is associated with a lower incidence of CPSP compared to patient-controlled intravenous analgesia (PCIA) after VATS.

## 2 Methods

### 2.1 Study design and participants

Ethical approval for this study (B2023-358) was provided by the Ethical Committee of Zhongshan Hospital, Fudan University, Shanghai, China (Chairperson Prof Jia Fan). This study was conducted in line with the STROCSS criteria [12]. We reviewed the medical records of patients admitted to our center between January 2020 and December 2022. The inclusion criteria were as follows: (1) age >18 years, (2) American Society of Anesthesiologists (ASA) classification I–III, (3) no previous VATS or open thoracic surgery, (4) no history of acute or chronic pain, (5) no history of narcotic addiction, and (6) elective VATS surgery. The exclusion criteria were as follows: (1) those undergoing postoperative rethoracic back surgery, (2) those with incomplete perioperative records, and (3) those who lost contact midway through the procedure.

### 2.2 Analgesia protocols

In this trial, participants were divided into two groups. The first group (PCIA group) underwent combined intravenous and inhalation anesthesia during surgery and postoperative pain management via intravenous self-controlled analgesia. The second group, the (epidural block [EPI] group) received combined intravenous and inhalation anesthesia during the procedure, followed by postoperative pain control using a combination of TEA and epidural self-controlled analgesia. Within the PCIA group, anesthesia induction was achieved using propofol at a concentration of 3–4 μg/mL and continuous infusion of remifentanil at 2–3 ng/mL, along with the administration of rocuronium bromide at a dose of 0.6 mg/kg. Intraoperatively, the participants received 20–40 μg of sufentanil, 1–2 mg of hydromorphone or 0.5–1 mg of oxycodone, and 500 mg of acetaminophen to maintain analgesia. Sedation was maintained by administering sevoflurane or desflurane at a concentration of 0.8–1 minimum alveolar concentration (MAC). Postoperatively, PCIA was used to deliver sufentanil 1 μg/mL or hydromorphone 0.04–0.05 mg/ml at a drip rate of 0.5–1 mL/h, with a single volume of 3–4 ml and an interval of 8 min between the doses.

In the EPI group, anesthesia induction involved the use of propofol at a concentration of 3–4 μg/mL; continuous infusion of remifentanil at 2–3 ng/mL, and administration of rocuronium bromide at a dose of 0.6 mg/kg. Intraoperatively, the participants received an injection of sufentanil at a dose of 20–40 μg. Additionally, to maintain analgesia, 0.2 % ropivacaine at a rate of 4–5 mL/h was administered through the epidural catheter. Anesthesia was maintained using sevoflurane or desflurane at a concentration of 0.8–1 MAC. Postoperatively, the patient received sufentanil 0.4 μg/ml or hydromorphone 8 μg/ml mixed with ropivacaine 1.2 mg/mL in a patient-controlled epidural analgesia pump (PCEA) at a drip rate of 3 ml/h, with a single dose of 3–4 ml and locking interval of 10 min.

The medications administered during the intraoperative period, duration of hospital stay, and time of drain removal were documented. In the ward, a standardized approach was adopted for ensuring analgesia in the two groups, involving the uniform intravenous administration of propacetamol at a dose of 1–2 g. Additionally, if the patient's numerical rating scale (VAS) score exceeded 4, they were administered oral aminophenol oxycodone (5 mg) or intravenous parecoxib (20 mg).

VATS can be categorized into uniportal, biportal, and triportal VATS. The surgeon determined the size and placement of the incision based on specific surgical circumstances. Intraoperative information and immediate postoperative pain data were also collected from the operative records.

### 2.3 Collection of CPSP information

A telephonic questionnaire (Supplementary file 1) was used to gather information on CPSP at 3 or 6 months postoperatively, and consisted of the following questions [13]:

1. Do you currently have pain related to your thoracic surgery?
2. If your answer to 1 is positive, did you suffer from pain now?
3. If your answer to 1 is positive, what was the worst pain intensity? Score it using a VAS (0 = no pain; 10 = worst pain imaginable)

In this study, patients with a VAS score of ≥1 at 3 or 6 months postoperatively were considered to have CPSP.

## 3 Statistical analyses

The primary outcome variable and statistical analysis plan were defined before data collection. The primary outcome variable in the study was chronic pain related to thoracic surgery at 3 or 6 months after surgery based on the following question: “Do you currently have pain related to your thoracic surgery?” Patients with chronic pain related to thoracic surgery at 3 or 6 months after surgery were compared with patients without such pain. Two-sided 95 % confidence intervals (CI) for the incidence of thoracic surgery-related chronic pain at 3 and 6 months after surgery were calculated according to the Clopper and Pearson method.

The normality of continuous data was tested using the Shapiro–Wilk test as well as by examining the quantile-quantile plot. Normally distributed continuous variables were presented as means ± standard deviation (SD), and univariately compared using the two-sample *t*-test. When the distribution was not normal, the variables were expressed as medians, along with the first (Q25) and third quartiles (Q75), and were compared using the Wilcoxon rank-sum test. Categorical data were presented as frequencies and percentages, and compared using the chi-square test or Fisher's exact test, where appropriate. Patients who responded to the phone interviews at 3 and 6 months were included in the analyses. The missing data points were not imputed. Repeated measures analyses were performed using mixed-effects models with unstructured covariance structures. Univariate and multivariate statistical analyses were performed using least absolute shrinkage and selection operator (LASSO) logistic regression, and models were compared using the LASSO approach.

Akaike's Information Criterion (AIC). The goodness-of-fit of the final multivariate logistic regression model was evaluated using the Hosmer–Lemeshow test, where a P-value less than 0.05 indicates a lack of fit. The area under the receiver operating characteristic curve (c-statistic) for the multivariate logistic regression model was calculated.

Statistical analyses were performed using R version 3.2.5 (The R Foundation for Statistical Computing, Vienna, Austria). Plots were created using the SigmaPlot version 12.5 (Systat Software, San Jose, CA, USA). R version 3.6.3 for “glmnet” package was used to perform the LASSO logistic regression.

## 4 Results

### 4.1 Patient characteristics

**Fig. 1:**
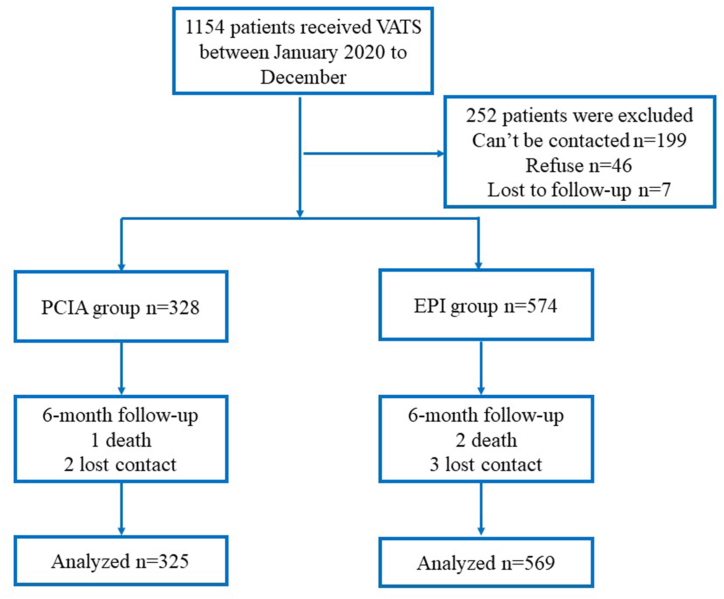
Flowchart of patient selection.

Overall, 1,154 individuals who underwent VATS at our hospital between January 2020 and December 2022 were assessed based on the aforementioned screening criteria. Among them, 203 could not be followed up and 57 declined to participate in the study. Eventually, 894 patients completed the follow-up interviews at 3 and 6 months, with 325 patients in the PCIA group and 569 in the EPI group (Fig. 1).

Patient characteristics and perioperative parameters are summarized in Table 1. Notably, the PCIA group exhibited a relatively younger age, higher proportion of women, greater utilization of intraoperative morphine, and a shorter duration of surgery than the EPI group. In the PCIA group, a larger proportion of patients underwent a uniportal incision approach, experienced a shorter hospital stay, and had a shorter period of chest tube retention.

**Table 1:** Baseline epidemiological and preoperative clinical characteristics.

	All patients (N = 894)	PCIA (N = 325)	EPI (N = 569)	P
**Age**				<0.001
≤40yr	135(15.10)	68 (20.9 %)	67 (11.8 %)	
41-≤65yr	502(56.15)	178 (54.8 %)	324 (56.9 %)	
>65yr	257(28.75)	79 (24.3 %)	178 (31.3 %)	
**Sex**				0.098
Female	522(58.39)	202 (62.2 %)	320 (56.2 %)	
Male	372(41.61)	123 (37.8 %)	249 (43.8 %)	
**BMI**	23.45 ± 3.14	23.2 (21.3–25.4)	23.2 (21.2–25.4)	0.759
**Lymphadenectomy**				0.006
No	377(42.17)	117 (36 %)	260 (45.7 %)	.
Yes	517(57.83)	208 (64 %)	309 (54.3 %)	.
**ASA**				0.381
1	318(35.57)	121 (37.2 %)	197 (34.6 %)	.
2	565(63.20)	202 (62.2 %)	363 (63.8 %)	.
3	11(1.23)	2 (0.6 %)	9 (1.6 %)	.
**Cardiovascular**				0.703
No	478(53.47)	177 (54.5 %)	301 (52.9 %)	
Yes	416(46.53)	148 (45.5 %)	268 (47.1 %)	
**Tumor**				0.002
No	861(96.31)	177 (54.5 %)	301 (52.9 %)	.
Yes	33(3.69)	148 (45.5 %)	268 (47.1 %)	.
**Respiratory**				0.898
No	867(96.98)	316 (97.2 %)	551 (96.8 %)	.
Yes	12(3.02)	9 (2.8 %)	18 (3.2 %)	.
**Diabetes mellitus**				0.480
No	755(85.80)	279 (85.8 %)	477 (83.8 %)	.
Yes	125(14.20)	46 (14.2 %)	92 (16.2 %)	.
**Morphine** **Equivalent, mg**	10.00(5.00, 15.00)	15.0 (15.0–17.5)	10.0 (5.0–10.0)	<0.001
**Anestesia** **Duration, hours**	1.5(2.00, 2.5)	2.0 (1.5–2.5)	2.5 (2.0–2.5)	<0.001
**Operative time, hours**	1.3(1.8, 2.2)	1.9(1.4–2.3)	2.4(1.9–2.3)	<0.001
**Blood loss(ml)**	88.5(75.6, 92.5)	88.9(76.9, 95.2)	86.5(72.7, 91.3)	0.903
**Type.of.surgery**				<0.001
One	580(64.95)	280 (86.2 %)	300 (52.8 %)	.
Two	63(7.05)	19 (5.8 %)	231 (40.7 %)	.
Three	250(28.00)	26 (8 %)	37 (6.5 %)	.
**Time to pull out the chest drain, days**	4.00(3.00, 4.00)	3.00(3.00, 4.00)	4.00(3.00, 5.00)	<0.001
**Hospital stays, days**	6.00(5.00, 7.00)	5.00(4.00, 6.00)	6.00(5.00, 8.00)	<0.001
**NAS**				
**POD1d**	1.00(0.00, 3.00)	2.00(2.00, 3.00)	0.00(0.00, 2.00)	<0.001
**POD7d**	0.00(0.00, 1.00)	0.00(0.00, 1.00)	0.00(0.00, 1.00)	0.043

The data are given as mean ± SD, n (%), or median (interquartile range). BMI, Body Mass Index; ASA, American Society of Anesthesiologists; NAS, Numerical rating scale; POD, postoperative day.

### 4.2 Acute and chronic pain incidence in the EPI and PCIA groups

**Fig. 2:**
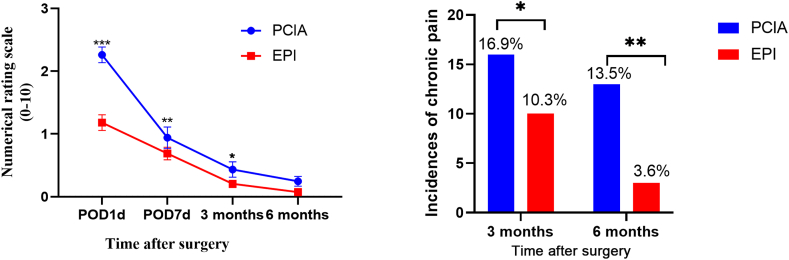
Numerical Rating Scale (VAS; 0–10) scores and incidence rates of chronic pain during the 6 months of the study separately for the PCIA and EPI groups. Left: VAS scores during the 6 months of the study separately for the PCIA and EPI groups. Note that patients with zero pain scores were included in this plot. When analyzed longitudinally, the time effect (P < 0.0001), analgesia effect (P < 0.0001), and their interaction (P < 0.0001) were statistically significant. Two-sided error bars represent standard deviation. Right: Incidence rates of chronic pain the PCIA and EPI groups at 3 and 6 months after surgery. POD: postoperative day, PCIA: patient-controlled intravenous analgesia, EPI: patient-controlled epidural analgesia. *P < 0.05 versus PCIA group, * *P < 0.01 versus PCIA group, and * * *P < 0.001 versus PCIA group.

The overall VAS score during the acute and chronic postoperative periods for all patients is plotted from the first postoperative day to 6 months postoperatively in Fig. 2 for both the EPI and PCIA groups. The average VAS score of the EPI group was lower than that of the PCIA group on postoperative day (POD) 1, POD 7, and at 3 months, whereas no significant difference was observed between the scores of the two groups at 6 months. The incidence rates of chronic pain in the PCIA group at 3 and 6 months were 16.9 % (55/325, 95 % CI: 9.3–32.7 %) and 13.5 % (44/325, 95 % CI: 8.7–33.4 %), respectively. The incidence rates of chronic pain in the EPI group at 3 and 6 months were 10.3 % (59/569, 95 % CI: 8.1–30.5 %) and 3.6 % (21/569, 95 % CI: 3.5–21.5 %), respectively. The incidence of chronic pain in the EPI group was significantly lower than in the PCIA group at both 3month(P = 0.0048) and 6month(P < 0.005) (Fig. 2).

### 4.3 VATS-related chronic pain

A comparison of the incidence rates of chronic pain between the PCIA and EPI groups is presented in Table 2. Out of the 325 individuals in the PCIA group, 55 (16.92 %) reported persistent pain in and around their surgical scars (with a VAS score ≥1). In contrast, in the EPI group, 61 (10.72 %) out of 569 individuals experienced chronic pain. On conducting unstratified response analysis, the relative difference (RD) (%) was calculated as −6.20, relative risk (RR) as 0.63, and odds ratio (OR) as 0.59. The Chi-square test yielded a P-value of 0.0079, whereas the Fisher's exact test yielded a P-value of 0.0096. Regarding the ASA scores and presence of underlying medical conditions, such as cardiovascular disease, respiratory disease, and diabetes, no statistically significant differences were observed between the two groups.

**Table 2:** Chronic pain Analysis.

	PCIA	EPI
	(N = 325)	(N = 569)
Chronic pain	55.00 (16.92 %)	61.00 (10.72 %)
Unstratified Response Analysis		
Risk Difference, RD (%) (95 % CI)		−6.20 (−11.01–−1.40)
Risk Ratio, RR (95 % CI)		0.63 (0.45–0.89)
Odds Ratio, OR (95 % CI)		0.57 (0.39–0.85)
p-value (Chi-squared test)		0.0059
p-value (Fisher's exact test)		0.0096

**Fig. 3:**
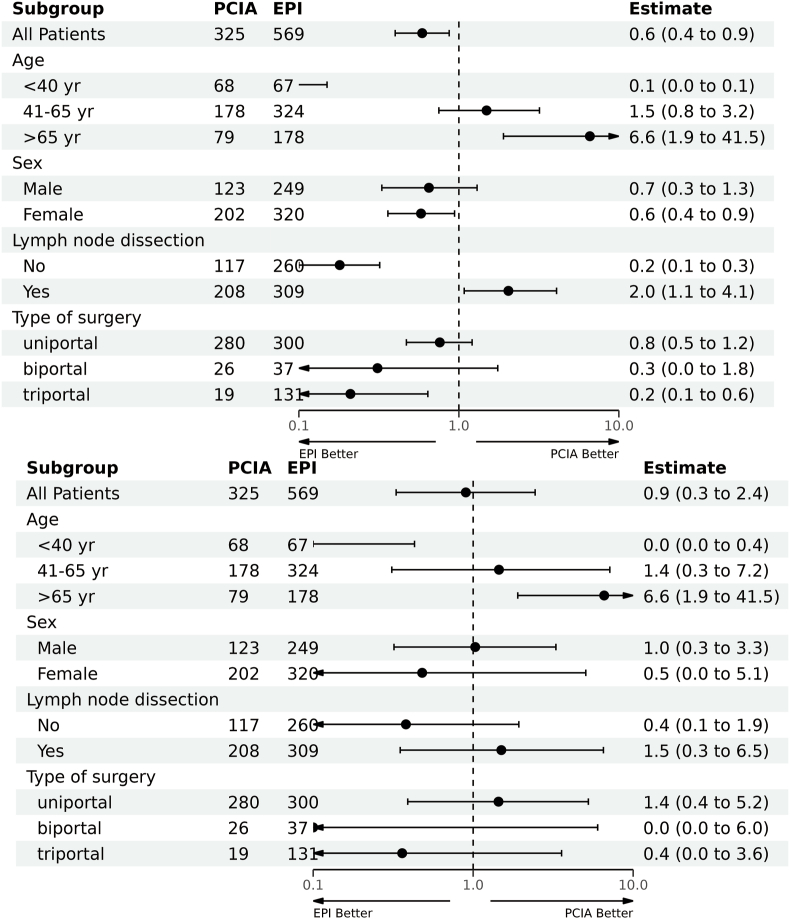
Forest plot for the interaction between preselected baseline factors and the effect of PCIA and EPI alone on chronic pain. The estimated overall odds ratio was derived from univariate and multivariable logistic regression models adjusted for age, sex, hospital stays, VAS score at postoperative day 1 (VAS1), and VAS3. In the subgroup analyses, we assessed the treatment-by-covariate interaction with the primary outcome, adjusting for the same baseline variables. PCIA: patient-controlled intravenous analgesia, EPI: patient-controlled epidural analgesia, VAS, Numerical Rating Scale.

Logistic regression analysis with LASSO variables was used to further examine the incidence of CPSP (Table 3). Age, sex, body mass index (BMI), hospital stay, analgesia type, and acute post-surgical pain were found to be related to chronic pain. After adjusting for age, sex, BMI, length of hospital stay, VAS at postoperative day 1(VAS1), and VAS3, the OR was 0.90 (95 % CI: 0.33–2.44; P = 0.03; Table 4). Univariate analysis showed that age and lymph node dissection were associated with chronic pain, and there was a higher incidence of chronic pain in patients with lymph node dissection and in older patients, whereas multivariate analysis results revealed that chronic pain was only related to age in the PCIA and EPI groups (Table 5). To gain a deeper insight into the variations in these factors between the two groups, a subgroup analysis was conducted. Subgroup interactions were statistically significant for age and lymph node dissection. However, after adjusting for age, sex, BMI, length of hospital stay, VAS1, and VAS3, no subgroup interactions remained statistically significant (Table 5 and Fig. 3).

**Table 3:** Logistic regression with variable reduction (variables Selected by Regularized Regression(Lasso)).

	OR (univariable)	OR (multivariable)
Age		
<40	–	–
41-65	0.16 (0.10–0.25, p < 0.001)	0.30 (0.11–0.80, p = 0.018)
>65	0.22 (0.13–0.37, p < 0.001)	0.43 (0.13–1.40, p = 0.162)
Sex		
Female	–	–
male	0.65 (0.42–0.97, p = 0.039)	0.21 (0.08–0.50, p = 0.001)
BMI	0.95 (0.89–1.01, p = 0.086)	0.93 (0.82–1.06, p = 0.289)
lymph node dissection		
No	–	–
Yes	0.50 (0.34–0.75, p = 0.001)	–
ASA		
1	–	–
2	0.70 (0.47–1.04, p = 0.075)	–
3	0.54 (0.03–2.89, p = 0.556)	–
Cardiovascular and cerebrovascular history		
No	–	–
Yes	0.64 (0.43–0.95, p = 0.029)	–
History of diabetes		
No	–	–
Yes	0.66 (0.34–1.17, p = 0.179)	–
Tumor history		
No	–	–
Yes	0.92 (0.27–2.40, p = 0.882)	–
Respiratory history		
No	–	–
Yes	0.83 (0.20–2.44, p = 0.770)	–
Morphine equivalent	1.05 (1.01–1.08, p = 0.010)	–
Anestesia duration, hours	1.09 (0.82–1.42, p = 0.543)	–
Operative time, hours	1.08 (0.77–1.43, p = 0.552)	
Blood loss(ml)	1.33 (0.88–2.31, p = 0.554)	
Type of surgery	one	–
uniportal	–	–
biportal	0.63 (0.24–1.40, p = 0.300)	–
triportal	0.70 (0.43–1.10, p = 0.127)	–
Time to pull out the chest drain, days	0.98 (0.80–1.19, p = 0.813)	–
Hospital stays, days	0.94 (0.83–1.06, p = 0.317)	0.85 (0.64–1.13, p = 0.268)
Analgesia		
PCIA	–	–
EPI	0.59 (0.40–0.87, p = 0.008)	0.89 (0.32–2.43, p = 0.818)
POD1	3.57 (2.88–4.55, p < 0.001)	1.33 (0.85–2.13, p = 0.216)
POD7	10.85 (7.25–17.65, p < 0.001)	11.64 (7.04–21.18, p < 0.001)

Performance of multivaiate model: Number in dataframe = 894, Number in model = 888, Missing = 6, AIC = 195.9, C-statistic = 0.985, H&L = Chi-sq(8) 2.99 (p = 0.935) BMI, Body Mass Index; ASA, American Society of Anesthesiologists; NAS, Numerical rating scale; POD, postoperative day; PCIA, patient-controlled intravenous analgesia; EPI, epidural anesthesia.

**Table 4:** Logistic regression for chronic pain analysis.

	OR_1_	95%CI_1_	P
Unadjusted			
PCIA	–	–	
EPI	0.57	0.39, 0.85	0.006
Adjusted^a^			
PCIA	–	–	
EPI	0.90	0.33, 2.44	0.03

1OR = Odds Ratio, CI = Confidence Interval.

a Adjusted for age, sex, hospital.stays, POD1, POD7; PCIA, patient-controlled intravenous analgesia; EPI, epidural anesthesia.

**Table 5:** Subgroup analysis.

	Univariate Logistic Model					Multivariate Logistic Model					
	PCIA	EPI	OR(95%CI)	P	P for interaction	PCIA	EPI	OR(95%CI)	P	P forinteraction	
Overall	325 (100 %)	569 (100 %)	0.59 (0.40–0.87)	0.008		325 (100 %)	569 (100 %)	0.90 (0.33–2.44)	0.829		
Age					<0.001						0.05
<40	68 (21 %)	67 (12 %)	0.06 (0.02–0.15)	<0.001		68 (21 %)	67 (12 %)	0.03 (0.00–0.43)	0.03		
41-65	178 (55 %)	324 (57 %)	1.49 (0.75–3.19)	0.275		178 (55 %)	324 (57 %)	1.45 (0.31–7.15)	0.634		
>65	79 (24 %)	178 (31 %)	6.59 (1.90–41.55)	0.012		79 (24 %)	178 (31 %)	2.45 (0.23–49.86)	0.487		
Sex					0.795						0.576
Female	202 (62 %)	320 (56 %)	0.58 (0.36–0.94)	0.027		202 (62 %)	320 (56 %)	1.03 (0.32–3.29)	0.956		
male	123 (38 %)	249 (44 %)	0.65 (0.33–1.30)	0.214		123 (38 %)	249 (44 %)	0.48 (0.04–5.06)	0.539		
lymph node dissection					<0.001						0.27
No	117 (36 %)	260 (46 %)	0.18 (0.10–0.32)	<0.001		117 (36 %)	260 (46 %)	0.38 (0.07–1.93)	0.254		
Yes	208 (64 %)	309 (54 %)	2.04 (1.08–4.08)	0.034		208 (64 %)	309 (54 %)	1.50 (0.35–6.52)	0.578		
type of surgery					0.084						0.449
uniportal	280 (86 %)	300 (53 %)	0.76 (0.47–1.21)	0.243		280 (86 %)	300 (53 %)	1.44 (0.39–5.25)	0.58		
biportal	26 (8.0 %)	37 (6.5 %)	0.31 (0.04–1.75)	0.202		26 (8.0 %)	37 (6.5 %)	0.00 (0.00-Inf)	1		
triportal	19 (5.8 %)	231 (41 %)	0.21 (0.07–0.64)	0.004		19 (5.8 %)	231 (41 %)	0.36 (0.04–3.57)	0.372		

OR = Odds Ratio, CI = Confidence Interval.

**Fig. 4:**
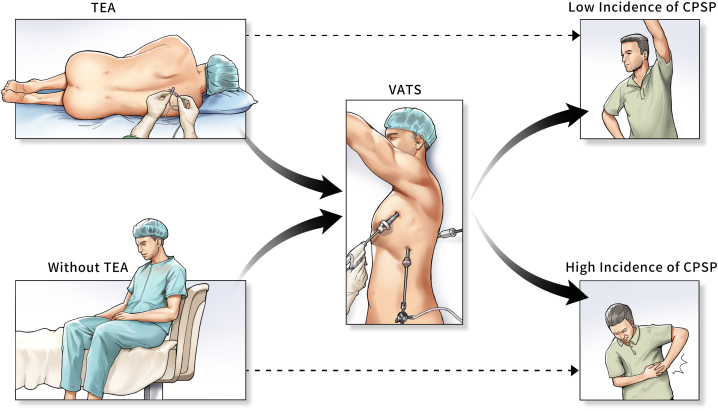
The sketch map of epidural anesthesia and analgesia on the incidence of CPSP after VATS.

The sketch map of epidural anesthesia and analgesia on the incidence of CPSP after VATS (Fig. 4).

## 5 Discussion

CPSP is common after thoracic surgery and may persist for a long time postoperatively. Even with the less invasive VATS, the incidence of CPSP remains high. Considering that the effective management of postoperative pain can decrease the incidence of CPSP as well as the superior pain relief effect of TEA, we investigated whether TEA could decrease the incidence of CPSP after VATS. The results indicated that compared to PCIA, TEA significantly reduced the incidence of CPSP after VATS.

Acute post-surgical pain is a risk factor for CPSP, and the incidence and severity of CPSP have been positively correlated with acute post-surgical pain [14,15]. This may explain why the overall incidence of CPSP, as well as the incidence of acute pain, were lower in the EPI group in the present study. However, univariate analysis revealed that VAS1 and VAS3 were not associated with chronic pain at 3 and 6 months, indicating that a single acute postoperative pain episode may not predict CPSP. A previous study [16,17] demonstrated several risk factors for CPSP, including demographics, genetic susceptibility, complications, pain, and psychological factors related to the operation. Xiao et al. [18] also reported that postoperative acute pain cannot predict CPSP in patients undergoing cardiac surgery. They claimed that severe acute postoperative pain results from a combination of baseline susceptibilities and inadequate postoperative pain management, and therefore does not constitute a true “predictive” risk factor, although it is an early manifestation of CPSP outcomes. However, the retrospective design of the present study restricted the analysis of some factors, such as lacking the psychological factors of the patients. Accordingly, we attributed the lower incidence of CPSP in the EPI group at least partially to the non-analgesic effects of TEA, which include attenuation of the perioperative stress response, anti-ischemic effects, effects on gastrointestinal function, and a reduction in pulmonary complications as well as a positive impact on the coagulation system and postoperative inflammatory response [19].

According to previous studies [20,21], CPSP was defined as pain at the surgical site (VAS score ≥1) which the patient identified as being related to the surgery. None of the patients in the present reported moderate to serve pain (VAS score ≥4) at 3 and 6 months postoperatively. At 3 months postoperatively, chronic pain occurred in 16.92 % and 10.72 % of patients in the PCIA and EPI groups, respectively, but the overall incidence of CPSP was lower than that reported in previous studies [[22], [23], [24]]. This discrepancy may be attributed to the large number of uniportal VATS cases in the two groups, especially in the PCIA group, as clinicians prefer general anesthesia for patients undergoing uniportal surgery. It is clear that the pain intensity associated with the biportal and triportal methods of VATS was higher than that associated with the uniportal method. Although there was a higher proportion of biportal and triportal cases in the EPI group, due to effective pain management by TEA, the average pain scores of the EPI group were still lower than those of the PCIA group on POD 7 and 3 month.

In patients undergoing VATS, pain management results have been mixed to date. Two studies reported comparable pain scores among TEA, systemic analgesia [25],and PCIA [26],whereas another study demonstrated lower postoperative pain scores [27] when TEA was combined with general anesthesia. Interestingly, one report indicated that TEA did not affect the incidence of persistent postoperative pain after VATS [25]. However, in this study, 118 cases of VATS for primary spontaneous pneumothorax were enrolled, with only 22 cases in the EPI group; thus, the level of evidence was significantly affected by the small sample size.

The serious complications of TEA in patients undergoing thoracic surgery included epidural hematoma (0.034 %) and epidural abscess (0.068 %) [28]. In patients undergoing VATS, the incidence of nausea and vomiting in the EPI group was also reportedly very high compared to that in the control group [27]. The most common side effects of TEA are hypotension, urinary retention, and pruritus, with the incidence of technical failures being 7 % [29]. However, no cases of epidural hematoma or abscess were recorded in the EPI group in this retrospective study, and we did not collect information on the incidence of other side effects, which may be attributed to the limitations of the study design and main purpose of the present study.

Many single-shot regional analgesia techniques have been recommended for thoracic surgery cases, such as thoracic paravertebral block, fascial plane block, serratus anterior block (SAPB) and intercostal nerve block [9,10]. It has been reported that the continuous thoracic paravertebral block (TPVB) technique can achieve similar acute pain relief to continuous TEA in patients undergoing thoracotomy [30]. A study revealed that continuous TEA significantly reduced the incidence of CPSP at 3 and 12 months after thoracotomy and provided better acute pain relief than single-shot TPVB combined with intravenous analgesia [31]. Another randomized study indicated that compared to continuous TEA, the number of patients with at least moderate chest pain at 6 months was lower with continuous TPVB, but with high levels of uncertainty [32]. According to another study, there was no statistical difference in the development of CPSP between patients who received continuous TEA and TPVB [33]. However, no studies have investigated the pure effects of continuous TEA on CPSP compared to those of general anesthesia with PCIA. Huang et al. reported that the continuous analgesia method of paravertebral space catheterization cannot replace continuous epidural analgesia with regard to the puncture failure rate and analgesic effects in VATS, and TEA is still considered the gold standard [11]. After a single manual bolus injection of TPVB, a large volume of the local anesthetic can slowly spread in the rostral and caudal directions due to gravity, while continuous pumping of local anesthetics in the paravertebral space cannot achieve widespread blockade, which might be related to the wide and loose paravertebral space that laterally reaches the angulus costae [34]. Helms et al. reported that continuous analgesia via paravertebral space catheterization was ineffective [35]. Unlike the loose paravertebral space, the epidural space is relatively precise and narrow, and most local anesthetics are restricted to this space. Their effect is accurate and has been confirmed in long-term clinical applications. Based on these considerations, we investigated the effectiveness of continuous TEA after VATS in reducing the incidence of CPSP.

We did not investigate neuropathic pain in the present study, since it has been reported that it is not the major component of chronic postoperative pain after VATS [36]. Moreover, univariate analysis showed that there was a higher incidence of chronic pain in patients with lymph node dissection and older patients, which may be considered as high-risk cases. This study had several limitations. First, the currently available methods used to assess chronic pain are not precise. The pathogenesis of CPSP involves a multitude of factors, and relying solely on subjective assessments such as the presence or absence of pain, VAS scores, and VAS scores may not provide a comprehensive evaluation of CPSP. Developing objective assessments specifically related to pain could contribute to a standardized approach; however, such tools have not been developed yet. Moreover, pain management in CPSP is predominantly performed by thoracic surgeons in hospital wards and outpatient clinics. Hence, it is challenging to ascertain the ongoing fluctuations in pain and the correlation between oral medications and CPSP. Finally, because of the general limitations of retrospective studies, future prospective studies are needed to confirm the effects of TEA on CPSP in patients undergoing VATS.

## 6 Conclusion

In conclusion, the results of the present study indicate that compared to PCIA, TEA significantly decreased the incidence of CPSP after VATS. These results may be attributed to both the analgesic effects and non-analgesic effects of TEA, which is the gold standard for regional analgesia and a reliable method for patients undergoing VATS.

## Funding statement

This work was supported by 10.13039/501100001809National Natural Science Foundation of China (82271215).

## Data availability statement

Data will be made available on request.

## Declaration of patient consent

Informed patient consent was waived of by the ethics committee as the data was anonymized and the retrospective nature of the study.

## Additional information

None.

## CRediT authorship contribution statement

**Yiming Liu:** Investigation, Data curation. **Chenyu Wang:** Methodology, Formal analysis. **Zhixiang Ye:** Methodology, Formal analysis, Conceptualization. **Ling Jiang:** Writing – review & editing, Writing – original draft, Formal analysis. **Changhong Miao:** Validation, Supervision, Conceptualization. **Chao Liang:** Writing – review & editing, Writing – original draft, Supervision, Investigation, Conceptualization.

## Declaration of competing interest

The authors declare the following financial interests/personal relationships which may be considered as potential competing interests:Liang chao reports financial support was provided by 10.13039/501100001809National Natural Science Foundation of China. If there are other authors, they declare that they have no known competing financial interests or personal relationships that could have appeared to influence the work reported in this paper.
