# Supplementary material for: Effects of epidural anesthesia and analgesia on the incidence of chronic pain after thoracoscopic lung surgery: A retrospective cohort study
Source: Heliyon. 2024 Jul 31;10(15):e35436. doi: 10.1016/j.heliyon.2024.e35436 (PMC11334903; doi:10.1016/j.heliyon.2024.e35436)
Supplement: Multimedia component 1 [file mmc1.docx]

**Supplemental 1. Telephone Questionnaires for Chronic Pain in Patients after Video-Assisted Thoracic Surgery (VATS)**

**Questionnaire for Chronic Pain After VAST**

1. Do you currently have pain related to your thoracic surgery?

□ Yes □ No

2. If your answer to 1 is positive, did you suffer from pain now?

□ Yes □ No

3. If your answer to 1 is positive, what was the worst pain intensity? Score it using a numeric rating scale

(0 = no pain; 10 = worst pain imaginable)
